# Is there a role for combination anti-remodelling therapy for heart failure secondary to chronic rheumatic mitral regurgitation?

**DOI:** 10.5830/CVJA-2016-095

**Published:** 2017

**Authors:** Ruchika Meel, Ferande Peters, Elena Libhaber, Mohammed R Essop,

**Affiliations:** Division of Cardiology, Chris Hani Baragwanath Academic Hospital and University of the Witwatersrand, Johannesburg, South Africa; Division of Cardiology, Chris Hani Baragwanath Academic Hospital and University of the Witwatersrand, Johannesburg, South Africa; Division of Cardiology, Chris Hani Baragwanath Academic Hospital and University of the Witwatersrand, Johannesburg, South Africa; Division of Cardiology, Chris Hani Baragwanath Academic Hospital and University of the Witwatersrand, Johannesburg, South Africa

**Keywords:** heart failure, mitral regurgitation, combination therapy

## Abstract

**Introduction:**

The value of combination anti-remodelling therapy for heart failure (HF) secondary to mitral regurgitation (MR) is unknown. We studied the effect of anti-remodelling therapy on clinical and echocardiographic parameters in patients with severe chronic rheumatic mitral regurgitation (CRMR) presenting in HF.

**Methods:**

Thirty-one patients (29 females) at Chris Hani Baragwanath Academic Hospital, treated with combination therapy for HF due to CRMR and New York Heart Association functional class II–III symptoms, underwent prospective six-month follow up.

**Results:**

Mean age was 50.7 ± 8.5 years. No patients died or were hospitalised for HF during the study period. No worsening of clinical symptoms or functional status, or left and right ventricular echocardiographic parameters (p > 0.05) was noted. Peak left atrial systolic strain improved at six months (18.7 ± 7.7 vs 23.6 ± 8.5%, p = 0.02).

**Conclusion:**

This preliminary analysis suggests that combination anti-remodelling therapy may be beneficial for HF secondary to CRMR. We had no HF-related admissions or deaths, and no deterioration in echocardiographic parameters of ventricular size and function.

## Introduction

For patients with valvular disease, direct pressure or volume overload results in cardiac remodelling and eventually heart failure (HF).^1^-^3^ Changes in neuro-hormonal signalling and genotype result in abnormal structure and function of both the myocyte and interstitial space.^2^-^4^ In chronic mitral regurgitation (MR), the persistent volume overload of the left atrium and ventricle, after a period of compensation, results in myocardial dysfunction through these mechanisms.^5^ This eventually culminates in atrial fibrillation, HF and death if left untreated.^6^

At present, surgery is the mainstay of therapy for patients with symptomatic severe MR and markers of left ventricular (LV) systolic dysfunction.^7^ Surgery is associated with non-negligible morbidity and mortality rates, even in established centres, especially in patients with LV dysfunction and high New York Heart Association (NYHA) functional class.^8^,^9^

The use of medical therapy for chronic MR has been largely non-conclusive and controversial.10 Most were small studies involving angiotensin converting enzyme (ACE) inhibitors and beta-blockers in degenerative MR.^10^-^12^ Guidelines on valvular heart disease recommend medical therapy for HF (ejection fraction < 50%) in chronic MR (class IIa, level of evidence B).^7^

No study has systematically looked at the effects of combination anti-remodelling therapy (ACE inhibitors, betablockers, aldosterone receptor antagonist) in HF secondary to MR. There is proven mortality and morbidity benefit of combination anti-remodelling therapy in systolic HF as a result of ischaemia and cardiomyopathy.^13^-^15^

We hypothesised that a similar benefit may be derived in HF secondary to CRMR. This could potentially offer an alternative option to these patients who are at high risk for surgery or are not inclined to undergo surgical intervention. Furthermore, the benefit of anti-remodelling therapy may be extended to asymptomatic patients with significant MR to prevent disease progression and delay the time to surgery. We therefore aimed to study the effect of anti-remodelling therapy, including ACE inhibitors and beta-blockers, in terms of clinical outcome, and traditional as well as newer echocardiographic parameters, such as two-dimensional strain in patients with severe CRMR who presented with HF.

## Methods

This prospective, observational sub-study formed part of a larger study on CRMR at the Chris Hani Baragwanath Academic Hospital. Patients were enrolled between January and December 2014. The study was approved by the University of the Witwatersrand ethics committee (M140114). All patients were screened and those deemed to have severe CRMR and presented with HF were referred for possible inclusion in the study

HF was diagnosed as per the ACCF/AHA and ESC guideline definition.^15^,^16^ The assessment of HF was made based on a combination of the patient’s history, clinical signs as well as available clinical records. A total of 66 patients with presumed CRMR underwent clinical evaluation, resting electrocardiogram and detailed echocardiographic assessment according to a predetermined protocol.

The inclusion criteria were as follows: patients aged 18 years or older with echocardiographic features of severe CRMR; symptomatic (NYHA II–III); left ventricular ejection fraction ≤ 60%; refusing or awaiting surgery; and on medical therapy [ACE inhibitors, angiotensin receptor blockers (ARBs), beta-blockers or aldosterone receptor antagonist] for HF.

Patients were excluded if they had significant aortic valve disease, concurrent mitral stenosis (MS) with a valve area of less than 2.0 cm^2^, documented ischaemic heart disease, pre-existing non-valvular cardiomyopathy, prior cardiac surgery, congenital or pericardial disease, pregnancy, severe systemic disorders such as renal failure, uncontrolled hypertension (systolic blood pressure > 140 mmHg and diastolic blood pressure > 90 mmHg) on medication, or severe anaemia (haemoglobin < 10 g/dl).

Thirty-five patients were excluded due to the following: anaemia, renal dysfunction, mild or moderate MR, MR of non-rheumatic aetiology and inadequate image quality. The final sample included 31 patients. Most HF trials conducted with anti-remodelling agents required a minimum duration of three months to demonstrate benefit.^17^ We therefore followed up patients in this study for a period of six months.

All the patients included in the sub-study were receiving some form of medical therapy for HF. All were on the minimum dose of their respective HF medications and were up titrated at three months where indicated, based on symptoms, blood pressure, and urea and creatinine levels.

All patients enrolled in this study were on a combination of at least one anti-remodelling agent in addition to a diuretic for at least one week. Therapy comprised beta-blockers (atenolol, carvedilol), ACE inhibitors/ARBs (enalapril, perindopril, telmisartan) and an aldosterone receptor antagonist (spironolactone), in addition to digitalis and diuretics. Medication was initiated at the discretion of the treating physician. All medications were either down titrated or withdrawn, or substituted on follow-up visits if side effects were reported.

Patients were followed up at one, three and six months. At one and six months, a full clinical assessment was done, including the Minnesota HF questionnaire and six-minute walk test. The dose of the medication was titrated at one month and full titration was achieved at three months by the treating physician.

Transthoracic echocardiography was performed on all patients in the left lateral position by experienced sonographers using a S5-1 transducer on a Philips iE33 system (Amsterdam, the Netherlands). The images were obtained according to a standardised protocol at baseline and at the six-month follow up. The data were transferred and analysed offline using the Xcelera workstation (Philips). Echocardiographic measurements were done by the researcher at baseline and the follow-up measurements were done by an experienced sonographer who was blinded to the initial results.

For two-dimensional and Doppler quantification, all linear and volumetric chamber measurements were performed according to the American Society of Echocardiography (ASE) chamber guidelines at baseline and at six months. 18 Measurements relating to LV diastolic function were performed in accordance with the ASE guidelines on diastolic function and included pulse-wave Doppler at the mitral tips and tissue Doppler of both medial and lateral mitral annuli at baseline and at six months.^19^ Measurements relating to the right ventricle were based on the ASE guidelines on the right ventricle.^20^

MR was considered rheumatic in aetiology when the morphology of the valve satisfied the World Heart Federation (WHF) criteria for the diagnosis of chronic rheumatic heart disease.21 MR severity was assessed using qualitative, semiquantitative and quantitative methods (integrated approach) as per the ASE valvular regurgitation guideline.^22^,^23^ In equivocal cases, the echocardiographic data were integrated with the clinical evaluation by an experienced cardiologist to distinguish moderate from severe MR.

For speckle tracking echocardiography, left atrial peak systolic strain and left and right ventricular peak systolic strain were measured as previously described.^24^-^30^

## Statistical analysis

Statistical analysis was performed with Statistica (version 12.5, series 0414 for Windows). Continuous variables are expressed as mean ± SD or median (IQR). Paired Student’s t-test or Wilcoxon’s matched-pairs test were used to compare continuous variables. Categorical variables are expressed as percentages. A p-value of < 0.05 was recognised as statistically significant.

## Results

The baseline clinical characteristics are summarised in Table 1. There was no change in systolic and diastolic blood pressure or heart rate from baseline to six months [125 ± 12.6 vs 120.1 ± 10.2 mmHg, p = 0.09; 76.2 ± 12.2 vs 74.2 ± 11.02 mmHg, p = 0.5; 71.5 (70–81) vs 71.0 (61–80) beats/min, p = 0.43, respectively]. The median Minnesota HF score at the start and the end of treatment at six months was 34 (18–61) and 32.5 (13–48), respectively (p = 0.3). There was no difference in the six-minute walk test at the onset of treatment and at six months (265.5 ± 103.0 vs 275.4 ± 71 m, p = 0.6).

**Table 1:** Baseline clinical characteristics

*Variable*	*Number = 31*
Age (years)	50.7 ± 8.5
Gender (female/male)	29/2
Systolic blood pressure (mmHg)	125 ± 12.6
Diastolic blood pressure (mmHg)v	76.2 ± 12.2
Heart rate (beats/min)	71.5 (70–81)
Body surface area (m^2^)	1.73 ± 0.16
Body mass index (kg/m^2^)	28.1 ± 6.1
NYHA class II–III (%)	31 (100)
Hypertension (%)	29 (93)
HIV (%))	7 (23)
Atrial fibrillation (%)	2 (6.4)

Data are presented as median (interquartile range), mean ± SD or %. HIV, human immunodeficiency virus; NYHA, New York Heart Association functional class.

None of the patients were hospitalised for HF and all were alive at six months. Baseline and maximum therapeutic doses of their respective medications are summarised in Table 2. There was no worsening of left and right ventricular echocardiographic indices at baseline and at six months of therapy (Table 3).

Based on the integrated assessment (qualitative and quantitative parameters), MR severity did not change at the end of six months. No change in quantitative parameters of MR assessment was noted at the end of six months [(vena cava width: 6.5 ± 1.9 vs 6.0 ± 1.6 mm, p = 0.2; regurgitant fraction: 31.7 (18.9–57.7) vs 29.2% (15.7–53.5), p = 0.2)].

**Table 2:** Comparison between baseline and maximum medication dose of the study patients

*Medication*	*Number (%)*	*Baseline dose (mg)*	*Dose (mg) at six months*	*p-value*
Furosemide	30 (97)	75 ± 25.9	78.3 ± 34.9	0.67
Nifedipine XL	9 (29)	34.4 ± 21.8	47.7 ± 24.3	0.23
Digoxin	7 (23)	0.125	0.125	1.0
Enalapril	11 (35)	10 (2.5–20)	20 (10–20)	0.17
Perindopril	11 (35)	2.9 ± 1	4 ± 1.7	0.003
Carvedilol	29 (94)	12.5 (3.125–12.5)	50 (37.5–50)	< 0.001
Spironolactone	28 (90)	25 (12.5–25)	50 (50–75)	0.001

Data are presented as median (interquartile range), mean ± SD or %. Two patients were on telmisartan (40 mg at baseline and six months), and one was on atenolol (12.5 mg up-titrated to 25 mg at six months).

**Table 3:** Left and right ventricular echocardiographic parameters at baseline and six months of medical therapy

*Variable*	*Baseline (n = 31)*	*Six months of therapy (n = 31)*	*p-value*
Left ventricular indices			
LVEDD (mm)	55.5 ± 8.4	55.1 ± 8.0	0.8
LVESD (mm)	42.6 ± 9.4	40.7 ± 9.5	0.43
EDVi (ml/m^2^)†	81.8 (68.9–98.1)	79.1 (64.5–99.3)	0.6
ESVi (ml/m^2^)†	45.4 ± 17.8	42.8 ± 15.8	0.5
Ejection fraction (%)	46.9 ± 8.7	50.4 ± 10.1	0.1
E wave (cm/s)	113.2 ± 47.2	112.9 ± 42.1	0.9
A wave (cm/s)	102.0 ± 26.5	99.4 ± 28	0.7
E/A wave (ratio)	1.0 ± 0.4	1.1 ± 0.4	0.3
E´ medial (cm/s)	6.6 (4.5–8.2)	5.9 (4.7–7.8)	0.2
A´ medial (cm/s)	6.7 (5.1–8.2)	7.1 (5.9–8.3)	0.5
Medial E/E´(ratio)	18.5 ± 9.5	18.9 ± 9.7	0.8
Medial S´(cm/s)	6.1 ± 1.4	6.1 ± 1.2	1.0
LAVi (ml/m^2^)†	60.2 (47.1–89.4)	59.5 (44.2–82.4)	0.8
Right ventricular indices			
RV base (mm)	38.3 ± 6.25	35.8 ± 8.8	0.2
TAPSE (mm)	20.5 ± 2.9	20.6 ± 2.9	0.9
RV S´ (cm/s)	11.1 ± 2.7	11.6 ± 2.5	0.45
RAVi (ml/m^2^)†	26.5 (21.7–32)	24.7 (7.4–33.8)	0.6
PASP (mmHg)	33.2 ± 12.4	31.4 ± 11.5	0.5

Data are presented as median (interquartile range), mean ± SD or %. †Values are indexed to body surface area. EDVi, end-diastolic volume index; ESVi, end-systolic volume index; LAVi, left atrial volume index; EDD, end-diastolic diameter; ESD, end-systolic diameter; LV, left ventricle; PASP, pulmonary artery systolic pressure; RAVi, right atrial volume index; RV, right ventricle; TAPSE, tricuspid annular plane systolic excursion.

Left atrial peak systolic strain increased at six months (18.7± 7.7 vs 23.6 ± 8.5%, p = 0.02). However, no change in right and left ventricular peak systolic strain was noted at the end of combination therapy (–15.6 ± 5.0 vs –16.4 ± 5.9%, p = 0.56; –13.9 ± 4.3 vs –15 ± 4.0%, p = 0.28, respectively).

## Discussion

The main findings of this study were that patients with rheumatic MR and HF who were treated with optimal medical therapy had good short-term clinical outcomes. There was no deterioration in echocardiographic parameters of cardiac performance, and there was an improvement in left atrial peak systolic strain.

Some older, pre-echocardiographic studies on rheumatic heart disease in Western populations demonstrated variable natural histories, ranging from CRMR being a benign lesion with a normal life expectancy, to it being a severe, progressive and ultimately fatal disease.^31^-^33^ Natural history studies in degenerative MR, with follow up ranging from seven months to 10 years, have shown increased risk of sudden cardiac death and increased postoperative morbidity and mortality rates in the presence of severe MR symptoms, arrhythmias, left ventricular end-systolic dimensions (LVESD) ≥ 45 mm and ejection fraction (EF) ≤ 60%.^8^,R34-39 These studies, primarily pertaining to degenerative, significant MR, evaluated symptomatic and asymptomatic patients. Most of them concluded that with conservative management (medical therapy), outcomes were worse regarding cardiac death, progression to worsening NYHA functional class, left ventricular dysfunction, HF, atrial fibrillation and pulmonary hypertension.

Mũnoz et al.40 compared 29 patients with MR on medical therapy alone to 45 patients who underwent mitral valve replacement. They found at five-year follow up, a lower survival rate, faster progression to higher NYHA class, and more complications such as HF and atrial fibrillation in the medical therapy group.40 The main shortcomings of all these studies are the inclusion of mostly asymptomatic patients with significant MR, and the medication and dosages used were not systematically documented.

The subset of patients we followed up had mostly stage D heart failure due to organic valvular heart disease. They were on varied combination anti-remodelling therapy as part of their management. This provided us with the opportunity of observing this subgroup. The lack of change in left and right ventricular structural and functional indices may possibly be explained by the short duration of follow up, the disease-stabilising effect of anti-remodelling therapy, and/or the relatively younger age of our patients compared to degenerative MR patients. A lack of difference in MR severity, even after controlling the systolic blood pressure in our study, may be explained by the small effect of change in pressure gradient on regurgitant volume, static left ventricular volumes, and the rheumatic nature of the disease, whereby the orifice is fixed and not dynamic, as in degenerative MR.^5^,^12^,^41^

There was no change in left and right ventricular longitudinal strain parameters in our study and this may be attributed to the short duration of follow up. The reason for marked improvement in left atrial peak systolic strain may be that the left atrium remodels and recovers earlier than the ventricles after an injury, as shown by Therkelsen et al.^42^ Additionally, left atrial reverse remodelling has been known to occur independently of left ventricular reverse remodelling, due to the direct effect of drugs that inhibit the renin–angiotensin–aldosterone system (RAAS).^4^ Also, left atrial strain may be a more sensitive marker for detecting reverse remodelling than left atrial volumes, as noted in this study.

There are a number of studies that have evaluated the effects of individual drugs in degenerative MR. Most of these involved beta-blockers or vasodilators.^12^,^43^ The results from these mostly small, non-randomised trials were inconclusive.^10^,44 The effect of aldosterone receptor antagonists has not been evaluated in organic MR in humans. Additionally, no trial has systematically explored the effects of combination therapy (with or without HF), secondary to CRMR.

In our study, lack of benefit from individual agents may have been due to incomplete blockade of the sympathetic nervous system (SNS) or the RAAS. An example would be that of aldosterone escape during prolonged ACE inhibitor therapy.^45^ Other possibilities include activation of the kallikrein–kinin system due to an increase in bradykinin, which in turn activates matric metalloproteinases, resulting in collagen loss, a process that may be exacerbated by the inhibition of angiotensin II by ACE inhibitors.^46^

These drugs in combination have synergistic action; for example the combination of an ACE inhibitor, beta-blocker and aldosterone receptor antagonist suppresses myocardial fibrosis in systolic heart failure.^3^ Therefore, combination therapy with drugs that block the SNS and RAAS systems may be the answer. Most of our patients were on a combination of carvedilol, spironolactone and an ACE inhibitor. However, their effect on left ventricular function and rheumatic MR severity remains questionable.

All the patients however remained stable on combined medical therapy and none was hospitalised for HF or died during the six months of follow up. This is a relevant finding as more than 50% of patients with all-cause systolic HF are re-hospitalised within six months of HF assessment.^47^ The lack of sudden cardiac death and HF-related deaths in this study may be attributed to medical treatment, or perhaps chance, due to the small sample size. Combined medical therapy may serve to stabilise the disease process, probably via neuro-hormonal modulation (likely the most important compensatory and deleterious mechanism in MR).^48^ Combination HF therapy may therefore serve as a bridge to surgery, given the long delays due to resource limitations, or as a substitute for surgery where patients decline surgery or have a high surgical risk due to severe ventricular dysfunction.

There were several limitations to this observational study. We had no control arm, there was a varied combination of medications, the exact duration of therapy at baseline was not clear due to incomplete records, the study subjects and the researchers were not blinded to the treatment, it was a small sample size, and the follow-up period was short.

## Conclusion

We have shown that combination anti-remodelling medical therapy in CRMR may be beneficial to prevent hospitalisation for HF and death. It may have a stabilising effect on HF secondary to chronic rheumatic MR. Further larger studies are needed to test the effect of combination therapy on chronic organic MR.
